# The Healer’s Wound: Personal Injury, Identity, and Ethical Responsibility in Orthopedic Surgery

**DOI:** 10.7759/cureus.115092

**Published:** 2026-08-24

**Authors:** Joseph Salem-Hernández, Roberto Cardona-Quiñones, Saidy A Salem-Hernández, Norman Ramírez, Rafael Fernandez-Soltero, Jose Bossolo-Flores

**Affiliations:** 1 Department of Orthopedic Surgery, Ponce Health Sciences University, Ponce, PRI; 2 Department of Neurology, VA Boston Healthcare System, Boston, USA

**Keywords:** jungian psychology, orthopedic surgery, peer support, physician as patient, professional identity, return to work, shadow integration, surgeon burnout, surgical ethics, wounded healer

## Abstract

Orthopedic surgeons dedicate their careers to treating musculoskeletal injury, yet when they become patients, professional culture discourages disclosure, treats help-seeking as weakness, and provides no structured pathway to care without career consequence. This narrative review examines the ethical dimensions of surgeon injury through two complementary concepts drawn from Jungian psychology: the wounded healer archetype and shadow integration. We searched PubMed, Scopus, and Google Scholar from January 1975 to May 2026 for literature on the physician as patient, occupational injury and burnout in orthopedic populations, help-seeking behavior, peer support after adverse events, and the wounded healer archetype, and we selected sources on the basis of relevance to the ethical questions addressed here rather than by formal quality appraisal. The literature describes two divergent trajectories. Where personal suffering is acknowledged and integrated, it is associated with higher measured empathy and with reported changes in how clinicians listen and tolerate uncertainty. Where it is suppressed, the pattern we describe as 'shadow projection' may follow, with distorted clinical judgment, reduced empathy toward patients reporting pain, and ethical failures that credentialing systems are not designed to detect. This second trajectory is offered as a conceptual interpretation and a hypothesis for testing, not as an empirically demonstrated causal pathway. Two fictional composite vignettes, constructed for illustration and not drawn from any identifiable surgeon, patient, or published case report, are used to make the two trajectories concrete. From this synthesis we derive an ethical framework addressing four domains: disclosure to patients and institutions, the duty to operate, the timing of return to the operating room, and the reciprocal obligations institutions owe their surgeons. We also propose practical recommendations for departments, training programs, and professional societies. The central argument is not that Jungian psychology replaces clinical judgment but that it provides a vocabulary for the conditions under which clinical judgment may fail silently.

## Introduction and background

Orthopedic surgery is the medical and surgical specialty concerned with the diagnosis, operative and nonoperative treatment, rehabilitation, and prevention of disorders and injuries of the musculoskeletal system, comprising bone, joint, ligament, tendon, muscle, and peripheral nerve. Its scope extends from acute trauma and fracture care to arthroplasty, spinal surgery, sports medicine, pediatric deformity correction, musculoskeletal oncology, and the long-term management of degenerative disease. The clinical need is large. Musculoskeletal disorders affect more than one billion people worldwide and are consistently among the leading global causes of years lived with disability [1]. The work itself is physically demanding, requiring sustained force, non-ergonomic posture, radiation exposure, and long operative hours [2].

The scenario that follows is a fictional composite constructed for illustration. It does not describe an identifiable person, and it is not drawn from a clinical record or from a published case report.

A 42-year-old orthopedic trauma surgeon sustains a distal radius fracture during a weekend skiing trip. As the emergency physician reviews the radiographs, the surgeon mentally catalogs fixation options, calculates time to union, and estimates return-to-operating-room timelines. Within days, he has consulted three colleagues, debated volar plating versus external fixation, and begun range-of-motion exercises weeks ahead of protocol. His treating hand surgeon recognizes the pattern: the surgeon-patient who cannot relinquish the medical self, who views injury as a career threat, and who negotiates healing as though it were a surgical problem to be solved rather than a human experience to be endured. He has not told his patients. He has not told his department chief. He has not asked for help.

This scenario illustrates a paradox at the heart of orthopedic surgery. Surgeons dedicate their careers to treating musculoskeletal injury, yet when they become patients, many struggle to accept care, to navigate role reversal, and to reconcile professional identity with vulnerability.

Injury among orthopedic surgeons is common rather than exceptional. In a survey of all orthopedic surgeons in Tennessee, 140 of 495 responded, and 61 of those respondents (44%) reported at least one workplace injury during their career; the anatomic regions most often involved were the hand (25%) and the lower back (19%), and more than a third of injured respondents reported that no institutional resources were available to support them during recovery [2]. In a larger cross-sectional survey of United States orthopedic surgeons, 1,645 respondents reported 2,702 work-related musculoskeletal injuries, of which 17.9% required operative treatment; of the 61 respondents who filed a disability claim, 66% returned to work and 34% retired early. In the same cohort, 55.8% reported psychological distress since beginning practice and 64.4% reported burnout [3]. Injury, in other words, is an occupational feature of the specialty, and a substantial minority of injured surgeons face it without institutional support.

Evidence that orthopedic surgeons find it difficult to accept care is more limited but consistent in direction. In a retrospective series of nine surgeons treated operatively for hand or wrist fractures, functional outcome was shaped not only by the injury but also by the surgeons’ personality, motivation, capacity to accept and adapt to injury, and the centrality of the hand to their careers. Most returned to operative duties ahead of the schedule set by their treating hand surgeons, at an average of 25 days after surgery, and many described the injury as a challenge to be overcome and subsequently modified their activities to protect the hand [4]. Reluctance to seek care is documented more broadly. In a national survey of 5,829 United States physicians, close to 40% reported that they would hesitate to seek professional help for a mental health condition because of concerns about repercussions for medical licensure [5]. In a cross-sectional survey of 622 members of the academic surgical community, 26.1% reported a previous mental health diagnosis, 15.9% screened positive for current depression, 18.4% for anxiety, 17.3% for post-traumatic stress disorder, and 11.0% for hazardous alcohol use, and screening positive was associated with substantially higher odds of suicidal ideation [6].

Burnout compounds this picture. A cross-sectional survey of 156 South African orthopedic surgeons and registrars, using the Stanford Professional Fulfillment Index, found an overall burnout rate of 72%, with registrars considerably more likely to be affected than consultants (odds ratio 5.68, 95% confidence interval 1.3 to 25.2) [7]. That figure sits at the high end of the published range. A systematic review of 34 studies, including 8,471 orthopedic surgeons, reported a mean burnout prevalence of 48.9%, with individual study estimates ranging from 15% to 90.4%, variation driven largely by differing instruments, thresholds, and national contexts [8]. Repeat sampling at a single United States institution found that burnout among orthopedic attendings and trainees was higher in 2023 than it had been in 2019, with excessive workload the strongest correlate across career stages [9].

Surgical complications wound the surgeon who performs them as well as the patient who sustains them. In qualitative telephone interviews with 11 consultant orthopedic surgeons across six high-volume National Health Service departments, analysis identified three themes: infection is at some point inevitable, yet surgeons still feel personally accountable; the emotional impact is profound; and surgeons rely on each other for support that is largely informal [10]. A separate qualitative study of Swedish orthopedic surgeons managing prosthetic joint infection described a comparable emotional burden and comparable reliance on ad hoc peer support, which suggests the finding is not confined to one health system [11].

There is also evidence that personal illness can enhance clinical capacity. In a mixed-methods study of 125 patients and 361 practitioners, doctors with a personal experience of illness scored significantly higher on a validated empathy scale than those without [12]. This finding is the empirical anchor for a much older idea.

Carl Jung used the term "archetype" to describe recurring, cross-cultural patterns of image and meaning that organize human experience and that appear in myth, dream, and clinical material [13]. Archetypes are not inherited content but recurring forms through which experience is structured. The wounded healer is one such pattern, expressed in the Greek figures of Chiron, the centaur whose incurable wound coexisted with his gift for teaching medicine, and Asclepius, the physician who was himself marked before he healed. Applied to clinical work, the archetype holds that the healer’s own wound, when consciously held, becomes a source of empathic understanding rather than a liability [14]. Groesbeck developed the clinical form of this idea, arguing that healing depends on the clinician’s contact with the wounded part of the self rather than on the projection of that wound onto the patient [15]. Contemporary work has qualified the archetype rather than simply endorsing it: a review of the wounded healer construct in psychotherapy found both potential benefit, in the form of deepened understanding and post-traumatic growth, and potential risk, in the form of impaired boundaries, compassion fatigue, and unaddressed impairment, and it identified stigma and self-stigma as the principal barriers to a clinician disclosing woundedness at all [16].

Jung paired the archetype with two further concepts. The shadow is the part of the personality that a person cannot consciously accept and therefore excludes from the self-image; it is a universal structural feature rather than a pathology, and its characteristic behavior is projection, in which the disowned quality is perceived instead as a deficiency in others. Individuation is the developmental process by which shadow material is recognized, tolerated, and integrated into a more complete sense of self through honest self-confrontation, usually with the help of another person. In the clinical setting, the analytic literature treats the clinician’s own unacknowledged woundedness as a determinant of how patient distress is perceived and managed [17].

Orthopedic surgery already has a precedent for embedding psychological frameworks in routine care. Psychosocial resiliency, meaning the modifiable set of cognitive, emotional, and behavioral resources that allow a person to adapt to pain, disability, and uncertainty, has been proposed as a legitimate target of orthopedic practice, delivered through integrated behavioral support colocated with surgical care [18]. The underlying rationale is that pain intensity and disability after musculoskeletal injury correlate more strongly with catastrophic thinking, kinesiophobia, and mood than with radiographic pathology [19]. The argument of this review is that the same infrastructure can be extended to the surgeons who deliver that care.

This narrative review addresses four questions. What ethical obligations arise when the healer is wounded? What does the injured surgeon owe patients, colleagues, and themselves? What does the orthopedic community owe its surgeons in return? And what practical structures would make an ethical answer possible? The Jungian framework is positioned throughout as a humanistic and interpretive lens that helps organize observations, not as a replacement for empirical evidence and not as a causal model. Alternative and competing explanations for the behaviors described, including economic pressure, liability exposure, staffing obligations, reputational concern, and institutional culture, are real and consequential. The Jungian reading offers one account of the psychological route by which those external pressures are internalized and acted upon.

## Review

Review methodology and literature search

This is a narrative review. We searched PubMed, Scopus, and Google Scholar from January 1975 to May 2026, with 1975 chosen because it marks the first substantial clinical treatment of the wounded healer archetype in the analytic literature. Search terms were combined across three domains: clinician as patient (physician as patient, surgeon injury, occupational injury, return to work, fitness for duty), clinician distress (burnout, moral distress, second victim, suicidal ideation, help-seeking, stigma, peer support), and conceptual framework (wounded healer, archetype, shadow, individuation, analytical psychology, medical humanities). Reference lists of included articles were hand-searched, and citation tracking was used to identify more recent work.

Sources were included when they reported empirical data on injury, illness, complications, or distress in surgical or orthopedic populations; when they described or evaluated an institutional response such as peer support, debriefing, or return-to-work assessment; or when they provided a primary conceptual account of the wounded healer, the shadow, or individuation. Sources were excluded when they addressed clinician well-being only in non-surgical populations without transferable content, when they were duplicate reports of the same dataset, or when they were non-peer-reviewed commentary without original argument. Studies were not formally appraised for risk of bias, and no quantitative synthesis was performed; both decisions follow from the heterogeneity of the evidence base and from the interpretive aim of the review. We drafted the review with reference to the six domains of the Scale for the Assessment of Narrative Review Articles, which cover statement of importance and aims, description of the literature search, referencing, presentation of evidence, and appropriateness of data use [20]. Selection was performed by two authors, and disagreements were resolved by discussion. We acknowledge that a search of this kind cannot exclude selective citation, and we return to this in the Limitations.

Summary of the evidence base

Table 1 summarizes the 14 principal sources on which the argument rests, recording for each the design, the main finding, and the specific role the finding plays in the argument developed below.

**Table 1:** Principal sources supporting the wounded healer framework in orthopedic surgery This table summarizes the sources that form the empirical and conceptual basis of the review. For each source, it records the design, the principal finding, and the specific role that finding plays in the argument developed in the text. The sources span epidemiological, quantitative survey, qualitative, systematic review, and conceptual methodologies. Together they support four claims: that occupational injury and psychological distress are common and frequently unsupported in orthopedic populations; that injury and surgical complications threaten professional identity and generate an unmet need for peer support; that burnout is highly prevalent and not improving; and that a personal illness history is associated with measurably higher empathy scores. Bracketed numbers correspond to the reference list. Note: Studies are listed in order of first citation in the text. Reference numbers are not consecutive because the table omits conceptual sources cited in the Introduction that do not report study findings.

Study	Design	Key finding	Relevance to argument
Safiri et al., 2021 [1]	Global Burden of Disease analysis, 195 countries	Musculoskeletal disorders are among the leading global causes of years lived with disability	Establishes the clinical domain and workload of the specialty
Davis et al., 2013 [2]	Cross-sectional survey, 140 of 495 orthopedic surgeons in one US state	44% reported at least one career workplace injury, most often hand (25%) and lower back (19%); over a third reported no institutional support resources	Injury is common and institutional support is frequently absent
Yakkanti et al., 2023 [3]	Cross-sectional survey, 1,645 US orthopedic surgeons	2,702 work-related musculoskeletal injuries, 17.9% requiring surgery; 34% of disability claimants retired early; 55.8% psychological distress; 64.4% burnout	Physical and psychological occupational harm co-occur
Chin et al., 1999 [4]	Retrospective case series, 9 surgeons with operatively treated hand or wrist fractures	Outcome shaped by personality, motivation, capacity to accept injury, and career centrality of the hand; most returned to operating ahead of schedule, average 25 days	Identity threat and career anxiety influence recovery and return decisions
Dyrbye et al., 2017 [5]	National survey, 5,829 US physicians, with review of licensure applications	Close to 40% would hesitate to seek mental health care because of licensure concerns	Disclosure carries a perceived professional penalty
Collins et al., 2024 [6]	Cross-sectional survey, 622 academic surgeons, trainees, and students	26.1% prior mental health diagnosis; 15.9% depression, 18.4% anxiety, 17.3% PTSD, 11.0% hazardous alcohol use; screening positive raised odds of suicidal ideation	Distress in the surgical workforce is prevalent and under-disclosed
O’Connor et al., 2022 [7]	Cross-sectional survey, 156 South African orthopedic surgeons and registrars	72% burnout on the Stanford Professional Fulfillment Index; registrars at higher risk (OR 5.68, 95% CI 1.3 to 25.2)	Burnout compounds vulnerability when surgeons become patients; trainees most exposed
Chahal and Matwala, 2025 [8]	Systematic review, 34 studies, 8,471 orthopedic surgeons	Mean burnout prevalence 48.9%, range 15% to 90.4%	Provides the international range within which single-country estimates should be read
Lu et al., 2025 [9]	Repeat cross-sectional survey, single US institution, 2019 and 2023	Burnout increased over the interval; excessive workload the strongest correlate at all career stages	The problem is not resolving over time
Mallon et al., 2018 [10]	Qualitative interview study, 11 NHS orthopedic consultants	Persistent personal accountability despite inevitability of infection, profound emotional impact, reliance on informal mutual support	Complications are psychological wounds; structured debriefing is an unmet need
Svensson et al., 2020 [11]	Qualitative interview study, Swedish orthopedic surgeons	Comparable emotional impact of prosthetic joint infection and comparable reliance on ad hoc peer support	The complication burden is not confined to one health system
Brady et al., 2015 [12]	Mixed-methods survey, 125 patients and 361 practitioners	Doctors with personal illness experience scored significantly higher on a validated empathy scale	Personal illness or injury may enhance empathic capacity
Hankir and Zaman, 2013 [14]	Case report and conceptual review	Wounded healer archetype applied to clinician illness narratives; narrative disclosure reduces stigma	Theoretical foundation for the framework
Zale et al., 2018 [18]	Perspective article, orthopedic practice	Psychosocial resiliency and integrated behavioural support recommended within orthopedic practice	Precedent for embedding humanistic frameworks in orthopedic care

The wounded healer archetype in orthopedic surgery

The wounded healer archetype describes clinicians whose personal suffering deepens therapeutic insight. In its original formulation, the healer’s wound is not a deficit but a source of understanding that enriches the clinical encounter, on the condition that the wound is consciously held rather than denied [14]. Groesbeck’s clinical elaboration made the mechanism explicit: when the clinician remains in contact with the wounded part of the self, the patient’s suffering is recognized rather than merely categorized; when that contact is lost, the wound is projected, and the clinician relates to an idealized image of the healthy expert instead of to the patient in front of them [15]. Anthropological work on the same tradition notes that the archetype has always carried a double edge, conferring authority on the healer while also imposing an obligation to remain in contact with vulnerability, and that modern medical training tends to preserve the authority while discarding the obligation [21].

The archetype is particularly resonant in orthopedics, where professional identity is closely bound to physical capability and technical mastery. Musculoskeletal injury does not merely impair the surgeon’s hand; it threatens what the surgeon takes themselves to be.

Contemporary data are consistent with the empathic claim, though the evidence is thinner than the claim’s rhetorical force suggests. In the mixed-methods study of 125 patients and 361 practitioners, doctors with a personal experience of illness scored significantly higher on a validated empathy scale; the published report describes the difference as statistically significant but does not report the magnitude of the difference, the instrument’s name, or an effect size, so the size of the association cannot be established from that source [12]. The most widely used validated instrument in this literature is the Jefferson Scale of Empathy, a 20-item self-report measure scored from 20 to 140 across three components, perspective taking, compassionate care, and standing in the patient’s shoes, with published national norms in the region of 110 to 117 for medical students and practitioners [22]. Readers should therefore treat the empathy finding as directionally supportive and quantitatively unspecified, and any future replication in orthopedic populations should report instrument, mean scores, and effect size.

Chin and colleagues documented the identity dynamic in a case series of nine surgeons who underwent operative fixation of hand or wrist fractures [4]. Functional outcome was shaped not only by the injury but also by the surgeons’ personality, motivation, capacity to accept and adapt to injury, and the centrality of the hand to their careers. Two features of that series are relevant here. First, most of the nine returned to operative duties ahead of the schedule their treating hand surgeons had set, at an average of 25 days after surgery, which indicates that timing decisions were being made by the injured surgeon rather than by the treating clinician. Second, the surgeons largely framed the injury as a challenge to be mastered and subsequently changed their behavior to protect the hand, which is closer to adaptive reframing than to integration in the sense used here and closer still to the professional identity the series describes: highly motivated individuals for whom career involvement is a principal source of self-worth. The series is small, uncontrolled, and now more than two decades old, and it should be read as a description of a pattern rather than as an estimate of prevalence. Economic pressure, liability exposure, and loyalty to patients are also operative in such decisions. These external pressures do not preclude a parallel intrapsychic dynamic; on the reading offered here, they are the material on which it acts.

Complications wound the surgeon who performs them as surely as injury does. In interviews with 11 orthopedic consultants managing prosthetic joint infection, surgeons described a persistent sense of personal accountability even where they accepted that infection cannot be wholly prevented, an emotional impact they characterized as profound, and support arrangements that were real but entirely informal [10]. The Swedish study reported a similar pattern, including active strategies of emotional management and a similar absence of formal structures [11]. Read alongside the empathy data, this literature suggests that clinicians who have themselves been patients report a lasting shift in how they relate to those in their care, including greater tolerance of uncertainty and a more nuanced understanding of what recovery demands [10-12].

The question is therefore not whether orthopedic surgeons sustain physical injury; the survey data establish that a substantial proportion do [2,3]. The question is whether the wounds that follow injury, complication, and cumulative distress are acknowledged and addressed or carried privately until they contribute to burnout, impairment, or worse.

Shadow, projection, and individuation: when the wound is denied

The counterpart of the wounded healer is the shadow. As set out above, the shadow is the excluded portion of the personality, universal rather than pathological, and its characteristic behavior is projection [17]. The process Jung called individuation, the conscious integration of shadow material through honest self-confrontation, is the alternative path.

The second scenario that follows is also a fictional composite constructed for illustration and does not describe an identifiable person.

A 51-year-old spine surgeon develops lumbar radiculopathy following a long operative day. He adjusts his standing position, increases his analgesic intake, and tells no one. He modifies his operative technique to spare his back rather than to optimize exposure. In clinic, he grows impatient with patients who rate their pain as severe after procedures he performed while managing his own. He does not see the irony. He sees weakness.

The proposed mechanism is psychologically coherent but not empirically demonstrated, and we advance it as a hypothesis. On this account, pain that is suppressed rather than integrated is projected outward so that what cannot be accepted in the self is perceived as deficiency in others [17]. A surgeon who endures injury in silence may come to use personal stoicism as an implicit clinical yardstick, with the result that patients reporting pain the surgeon has privately deemed manageable are judged, in that private calculus, to be insufficient. What might have become a bridge becomes a wall instead. The available literature supports the components of this account separately rather than the pathway as a whole: stigma and self-stigma reliably suppress clinician disclosure of woundedness [5,16]; distress is prevalent and under-reported in surgical populations [6]; and clinician distress is associated with reduced empathy and with self-reported medical error [6,9]. What has not been demonstrated is a causal chain running from suppressed personal suffering, through projection, to a specific documented failure of clinical judgment. Establishing that would require prospective designs of a kind that do not yet exist in this field.

The clinical concerns that follow from the hypothesis are best stated as theoretical risks rather than as observed outcomes. A surgeon operating through unacknowledged pain might modify technique, limit exposure, or make intraoperative decisions shaped by personal tolerance rather than by the patient’s anatomy; a surgeon self-prescribing analgesics to maintain function would have compromised clinical judgment in a way that no credentialing process is designed to detect. We are not aware of published data quantifying either of these specific intraoperative consequences in orthopedic surgery, and we do not claim them as established. They are, however, plausible enough and serious enough to justify the procedural safeguard we recommend below: that the distinction between discomfort, treatable pain, and functional impairment compromising operative safety be drawn precisely and by an independent clinician rather than by the surgeon in question.

Individuation describes a different trajectory. On the analytic account, the clinician who works consciously through injury becomes more present and better calibrated, and the wound becomes a source of clinical understanding rather than an unexamined bias [14,15]. Again, this is a conceptual claim consistent with the empathy literature [12] rather than a demonstrated outcome, and it is the reason we frame the recommendations below as reasoned proposals requiring evaluation.

Ethical dimensions

Disclosure and the Duty to Operate

The injured surgeon faces an immediate ethical question: what must be disclosed, and to whom? Non-maleficence is unambiguous where operative capacity is impaired, in which case disclosure and temporary withdrawal are required. A surgeon who cannot grip or pronate without pain should not perform procedures that demand those actions. Where capacity is preserved, disclosure to individual patients may reasonably be discretionary, since the relevant consideration for consent is the surgeon’s present ability to perform the procedure safely rather than their medical history in general. Transparency with institutional leadership is a different matter. It enables coverage planning, and it initiates support that the surgeon may be reluctant to request. Tanaka and colleagues, writing on the physician who becomes a patient, note that such physicians face competing obligations to self, profession, and institution, and that honest capacity assessment by an independent clinician is an ethical obligation rather than a courtesy [23].

The empirical literature explains why disclosure is nonetheless uncommon. Where close to 40% of physicians report that licensure questions would make them hesitate to seek mental health care, the reticence is a rational response to an institutional incentive rather than a character flaw [5]. Any ethical framework that asks surgeons to disclose without first changing that incentive structure places the burden in the wrong place.

Premature Return and the Culture of Stoicism

The pattern documented by Chin and colleagues, returning to operative duties ahead of the treating clinician’s schedule, reflects a broader cultural problem [4]. Burnout is highly prevalent, with a pooled estimate near 49% across 34 studies and rates as high as 72% in individual cohorts, and trainees are consistently among the most affected [7,8]. Repeat measurements at one institution found the problem worsening rather than resolving [9]. Surgeon distress is linked to serious mental health consequences: in the American College of Surgeons survey of suicidal ideation, ideation in the previous 12 months was substantially more common among surgeons than in the general population, and surgeons who reported ideation were markedly less likely to seek help because of concern about the effect on their license [24]. Burnout in orthopedic surgeons specifically has been characterized as a systems problem with individual manifestations, requiring organizational as well as personal remedies [25].

Where stoicism is valorized and vulnerability stigmatized, injured surgeons make decisions under professional duress, weighing recovery against career. This harms patients, because impaired surgeons continue to operate, and it harms surgeons, because each unaddressed wound raises the cost of the next. The failure is systemic, implicating training programs, department chiefs, credentialing bodies, and professional societies, all of which shape the incentives under which an individual surgeon decides whether to disclose [26].

The Duty to Self-Care and the Reciprocal Obligation of Institutions

Institutions have an ethical obligation to their surgeons, not only to their patients. Providing coverage, facilitating access to care, and normalizing help-seeking are obligations that follow from the same duty-of-care logic that governs patient treatment [26]. A department that improvises coverage during a crisis rather than planning for it in advance has failed preventably [23]. Psychosocial resiliency and structured behavioral support have already been advanced as embeddable components of orthopedic practice, with integrated behavioral clinicians colocated in surgical clinics, brief protocolized interventions targeting catastrophic thinking and fear of movement, and routine screening for psychological distress [18,19]. The same infrastructure can be extended to the surgeons who deliver that care, and doing so requires no new conceptual commitment, only a decision to include the clinician in the population served.

Complications, the Second Victim, and the Obligation to Debrief

The concept of the second victim, introduced to describe the clinician who is harmed by their own involvement in an adverse event, provides the established ethical vocabulary for the complication literature reviewed above [27]. Programmatic responses exist and are well described. Peer support programs that train clinicians to provide structured, confidential, non-judgmental outreach after adverse events have been implemented at scale in academic centers, and they are consistently preferred by clinicians over employee assistance referrals [28]. A specialty society program in pediatric surgery has demonstrated that such a model can be delivered across institutions rather than only within them [29]. Among trainees, facilitated discussion groups led by a therapist during protected time have proved feasible and acceptable, and participation in five or more sessions was associated with greater willingness to discuss personal problems with co-residents and greater reported peer support [30]. A scoping review of second victim syndrome among surgeons specifically found consistent evidence of emotional impact and a consistent shortfall in formal support [31]. The ethical argument is therefore not that debriefing might help, but that a known, feasible, and acceptable intervention is being withheld. Table 2 sets out the resulting framework across six ethical domains.

**Table 2:** Ethical framework for the orthopedic surgeon as patient This table translates the preceding analysis into a decision framework covering six ethical domains encountered by an injured or ill orthopedic surgeon. Each row pairs the practical question the surgeon or department faces with the governing bioethical principle, the action the authors recommend, and the references supporting that recommendation. The domains progress from immediate individual decisions, such as what to disclose and whether to continue operating, to institutional and cultural obligations, such as self-care and training culture. The framework is a deliberative aid derived from a narrative synthesis and expert reasoning; it is not a validated instrument and does not substitute for independent clinical or occupational health assessment. Note: Recommendations should be adapted to institutional policy and local legal requirements.

Ethical domain	Key question	Guiding principle	Recommended action	Reference
Disclosure to patients	Does impaired capacity require disclosure?	Non-maleficence; patient autonomy	Disclose and withdraw where operative capacity is impaired; discretionary where capacity is preserved	[4,23]
Disclosure to institution	Should leadership be informed of injury?	Duty of care; surgeon privacy	Communicate transparently with the department chief; institutional policy should protect privacy while activating coverage	[23]
Duty to operate	Is an injured surgeon obligated to continue operating?	Non-maleficence; beneficence	Honest capacity assessment required; patient safety precedes professional pride or financial pressure	[4,23]
Return-to-work timing	When is return to the operating room safe?	Evidence-based practice; patient safety	Independent occupational health assessment; graduated return; avoid self-determined premature return	[4]
Duty to self-care	Do surgeons have an obligation to maintain their own health?	Beneficence; sustainability of practice	Treat self-care as a professional obligation; seek psychological support after injury or complication	[10,18,28]
Training culture	Is perpetuating stoicism ethical?	Justice; professional formation	Normalize vulnerability; provide safe and confidential channels for trainees to report injury or distress; remove licensure and credentialing questions that penalize care-seeking	[5,14,16,30]

Toward a Culture of Healing Healers

The orthopedic surgeon as a patient occupies a paradoxical position: the healer who is wounded, the expert who becomes vulnerable, and the operator who must accept care. Personal injury, when met with institutional support and honest reflection, may deepen empathy, recalibrate clinical judgment, and support a more authentic relationship with patients. This transformation requires what the current culture does not reliably provide: permission to be unwell, access to care without professional penalty, and colleagues willing to ask the question Weil identified as the most important a person can put to another in distress, which is simply to ask what the other is going through [32].

Table 3 sets out six practical recommendations. Because the reviewer of any such list is entitled to know how it was ordered, we state the basis explicitly. Recommendations were ranked on two dimensions assessed by author consensus rather than by a formal appraisal instrument. The first is the strength of supporting evidence, graded high where the intervention has been implemented and evaluated in surgical or orthopedic populations with reported outcomes, and moderate where support is conceptual, extrapolated from non-surgical clinician populations, or limited to expert recommendation. The second is implementation barrier, graded low where the action requires a departmental or program-level decision and existing resources, and high where it requires cultural change, external regulatory change, or new funding. Items rated high priority combine graded evidence with a low implementation barrier. This ranking is a transparent expert judgment, not an evidence grade derived from a validated framework, and readers should weigh it accordingly.

**Table 3:** Practical recommendations for the orthopedic community This table sets out six actionable recommendations for departments, training programs, and professional societies. For each, it identifies the audience responsible for implementation, an assigned priority, a concrete first step, and the basis on which the recommendation and its priority rest. Recommendations are ordered from those combining graded supporting evidence with the lowest implementation barrier to those requiring longer-term cultural change. The table is intended for use as an institutional planning document alongside the ethical framework in Table 2. Note: Priority reflects author consensus on strength of supporting evidence and ease of implementation, as described in the preceding paragraph. High indicates that implementation is supported by existing evidence and can proceed with current resources; moderate indicates a beneficial but context-dependent measure requiring longer-term cultural change.

Recommendation	Target audience	Priority	Key action	Basis
Normalize help-seeking and disclosure	Departments, professional societies, licensing bodies	High	Establish confidential reporting pathways and peer support programs; remove career penalties and licensure questions that penalize seeking care	Implemented and evaluated peer support models; documented licensure-related deterrence [5,16,28]
Peer debriefing after complications and injuries	Senior surgeons, department chiefs	High	Provide structured facilitated debriefing following serious complications and personal injuries	Qualitative evidence of unmet need in orthopedics; established second victim programs [10,11,27-29,31]
Proactive organizational planning	Department chiefs, administration	High	Establish succession plans and coverage protocols before a surgeon becomes ill or injured; activate them transparently	Documented absence of institutional support at time of injury; expert recommendation [2,23]
Clear return-to-work protocols	Occupational health, department chiefs	High	Require independent functional assessment before return to the operating room; use graduated return-to-practice models	Documented self-determined early return; ethical requirement for independent assessment [4,23]
Psychosocial resiliency training	Residency and fellowship directors	High	Embed reflective practice, emotional regulation, and facilitated discussion groups in surgical training curricula	Feasibility and acceptability demonstrated in surgical trainees; existing orthopedic precedent [18,19,30]
Foster a culture of vulnerability	Senior surgeons, program directors	Moderate	Model openness about personal injury and recovery; challenge norms of stoicism in training	Conceptual and narrative evidence; requires cultural change over years [14,16]

Implications

Three implications follow for practice. First, capacity assessment after surgeon injury should be relocated from the injured surgeon to an independent clinician, ideally within occupational health, with graduated return as the default rather than the exception. This is the single change most directly supported by the evidence reviewed, since the documented pattern is that injured surgeons return early on their own authority [4]. Second, debriefing after serious complications and personal injuries should be scheduled rather than offered, because the barrier identified in the qualitative literature is not the absence of willing colleagues but the absence of a structure that makes the conversation routine and therefore safe [10,11]. Third, institutions that already fund integrated behavioral support for orthopedic patients should extend eligibility to their own clinicians, which is an administrative rather than a conceptual step [18,19].

For education, the implication is that reflective practice should be treated as a technical competency rather than an optional supplement, and that trainees, who carry the highest burnout burden, should have access to facilitated discussion groups within protected time [7,30]. For policy, the implication is that licensure and credentialing questions that ask about mental health history rather than current impairment should be revised, since they measurably deter the behavior the profession claims to want [5]. For research, the framework generates testable predictions, set out below.

Strengths

This review has three strengths. First, it brings together literature that is usually kept apart: the epidemiology of occupational injury in orthopedic surgery, the qualitative literature on surgeon distress after complications, the survey literature on burnout and help-seeking, and the analytic literature on the wounded healer. Second, it translates a conceptual framework into a decision aid, with an explicit statement of the principle, the recommended action, and the supporting reference for each ethical domain, which makes the argument auditable and criticizable rather than merely suggestive. Third, it separates what the evidence shows from what the framework proposes, marking the shadow projection pathway as a hypothesis throughout so that readers can accept the empirical synthesis while rejecting the interpretation if they choose.

Limitations

This is a narrative synthesis rather than a systematic review, and it carries the corresponding limitations. The search, although documented above, was not registered; no formal risk of bias assessment was applied, and selective citation cannot be excluded. The empirical base is heterogeneous and largely descriptive. The most directly relevant clinical study includes only nine surgeons and is more than 25 years old [4]. The qualitative work is drawn from two national contexts, both of them high-income European health systems [10,11]. The burnout prevalence data come from cross-sectional surveys subject to response bias, with response rates as low as 7% in the largest injury survey and instrument heterogeneity wide enough to produce a fivefold range in reported prevalence [3,8]. The empathy finding on which the positive trajectory depends is reported without an effect size [12]. No study to date has prospectively evaluated whether structured psychological support alters return-to-work decisions or patient outcomes in injured surgeons.

The Jungian framework is interpretive rather than falsifiable in its general form, and we advance it as a lens for organizing observations rather than as a causal model. Alternative explanations for the same behavior, including financial pressure, contractual obligation, liability concern, and simple professional habit, are not excluded by anything presented here. The vignettes are fictional composites written to illustrate the two trajectories and carry no evidential weight. Accordingly, the recommendations offered here should be read as reasoned proposals requiring prospective evaluation.

Future research directions

Four lines of work would move this field from proposal to evidence. First, prevalence and outcome data are needed on orthopedic surgeons as patients specifically, including time to return to operating, who made the return decision, and whether independent assessment occurred; a multicenter registry of surgeon injury, modeled on existing complication registries and with appropriate confidentiality protections, would answer these questions more reliably than repeated cross-sectional surveys. Second, the empathy association should be tested prospectively in orthopedic populations using a named instrument with reported effect sizes, comparing surgeons with and without a personal history of musculoskeletal injury and adjusting for career stage. Third, the interventions recommended in Table 3, structured debriefing, facilitated discussion groups, and graduated return protocols, should be evaluated with prospective designs measuring disclosure rates, time to appropriate return, burnout scores, and, where feasible, patient-level outcomes; the demonstrated feasibility of facilitated groups in surgical trainees makes a stepped-wedge implementation study realistic [30]. Fourth, the shadow projection hypothesis should be operationalized and tested rather than assumed: a study correlating surgeons’ own pain and injury history with their documented analgesic prescribing, patient-reported satisfaction with pain management, and scores on validated empathy and stoicism measures would provide the first direct test, and a null result would appropriately narrow the framework’s claims.

## Conclusions

The wounded healer archetype holds that suffering is not antithetical to healing and can be one of its sources. The surgeon with the fractured wrist, the consultant preoccupied by a complication, and the resident struggling silently with burnout each stand between two trajectories: projection of the wound onto patients, or its integration. The evidence reviewed here does not establish which trajectory a given surgeon will follow, and we do not claim that it does. What the evidence does establish is that injury, complication, and distress are common in orthopedic practice, that institutional support at the time of injury is frequently absent, and that surgeons routinely make return-to-work decisions that their own treating clinicians would not have sanctioned. Those findings are sufficient to justify structural change independently of the framework. Departments and training programs can supply that structure through confidential disclosure pathways, pre-established coverage plans, independent capacity assessment before return to the operating room, and routine debriefing after complications and injuries. Licensing and credentialing bodies can remove the questions that make disclosure costly. The surgeon who is given permission and structure to acknowledge a wound may in time offer patients something the credentialing system cannot confer, and the operative log cannot measure: a physician whose own encounter with suffering has made them more fully present and more capable of witnessing the suffering of others. Whether that is so is a hypothesis worth testing, and this review is intended to make the test possible.
